# Multidimensional Cognitive Behavioral Therapy for Obesity Applied by Psychologists Using a Digital Platform: Open-Label Randomized Controlled Trial

**DOI:** 10.2196/14817

**Published:** 2020-04-30

**Authors:** Meelim Kim, Youngin Kim, Yoonjeong Go, Seokoh Lee, Myeongjin Na, Younghee Lee, Sungwon Choi, Hyung Jin Choi

**Affiliations:** 1 Department of Biomedical Sciences Seoul National University College of Medicine Seoul Republic of Korea; 2 Department of Psychology Duksung Women’s University Ssangmun-Dong, Dobong-Gu Republic of Korea; 3 Noom Inc New York City, NY United States; 4 Department of Biomedical Systems Informatics Yonsei University College of Medicine Seoul Republic of Korea

**Keywords:** obesity, digital health care, cognitive behavioral therapy, mobile phone

## Abstract

**Background:**

Developing effective, widely useful, weight management programs is a priority in health care because obesity is a major health problem.

**Objective:**

This study developed and investigated a new, comprehensive, multifactorial, daily, intensive, psychologist coaching program based on cognitive behavioral therapy (CBT) modules. The program was delivered via the digital health care mobile services Noom Coach and InBody.

**Methods:**

This was an open-label, active-comparator, randomized controlled trial. A total of 70 female participants with BMI scores above 24 kg/m^2^ and no clinical problems besides obesity were randomized into experimental and control groups. The experimental (ie, digital CBT) group (n=45) was connected with a therapist intervention using a digital health care service that provided daily feedback and assignments for 8 weeks. The control group (n=25) also used the digital health care service, but practiced self-care without therapist intervention. The main outcomes of this study were measured objectively at baseline, 8 weeks, and 24 weeks and included weight (kg) as well as other body compositions. Differences between groups were evaluated using independent *t* tests and a per-protocol framework.

**Results:**

Mean weight loss at 8 weeks in the digital CBT group was significantly higher than in the control group (–3.1%, SD 4.5, vs –0.7%, SD 3.4, *P*=.04). Additionally, the proportion of subjects who attained conventional 5% weight loss from baseline in the digital CBT group was significantly higher than in the control group at 8 weeks (32% [12/38] vs 4% [1/21], *P*=.02) but not at 24 weeks. Mean fat mass reduction in the digital CBT group at 8 weeks was also significantly greater than in the control group (–6.3%, SD 8.8, vs –0.8%, SD 8.1, *P*=.02). Mean leptin and insulin resistance in the digital CBT group at 8 weeks was significantly reduced compared to the control group (–15.8%, SD 29.9, vs 7.2%, SD 35.9, *P*=.01; and –7.1%, SD 35.1, vs 14.4%, SD 41.2, *P*=.04). Emotional eating behavior (ie, mean score) measured by questionnaire (ie, the Dutch Eating Behavior Questionnaire) at 8 weeks was significantly improved compared to the control group (–2.8%, SD 34.4, vs 21.6%, SD 56.9, *P*=.048). Mean snack calorie intake in the digital CBT group during the intervention period was significantly lower than in the control group (135.9 kcal, SD 86.4, vs 208.2 kcal, SD 166.3, *P*=.02). Lastly, baseline depression, anxiety, and self-esteem levels significantly predicted long-term clinical outcomes (24 weeks), while baseline motivation significantly predicted both short-term (8 weeks) and long-term clinical outcomes.

**Conclusions:**

These findings confirm that technology-based interventions should be multidimensional and are most effective with human feedback and support. This study is innovative in successfully developing and verifying the effects of a new CBT approach with a multidisciplinary team based on digital technologies rather than standalone technology-based interventions.

**Trial Registration:**

ClinicalTrials.gov NCT03465306; https://clinicaltrials.gov/ct2/show/NCT03465306

## Introduction

One of the major concerns of the health care industry is to find effective and widely practical solutions for weight management, given that obesity is one of the dominant public health problems of the 21st century. It is well known that weight reduction is highly correlated with reductions in the incidence of type 2 diabetes, as well as other medical weight-related comorbidities and psychosocial issues, and that it improves the quality of life [1].

Accordingly, various types of treatments for obesity have been developed. Several drugs have been proposed as pharmacotherapy for obesity since the 1990s, but most have demonstrated a lack of efficacy and unfavorable risks [2]. Bariatric surgery is another obesity treatment that has been used for over 50 years. Because the prevalence of obesity is rapidly rising, the number of patients who believe that bariatric surgery is an effective treatment to cure their obesity is also increasing [3]. Additionally, patients may believe that surgical intervention to overcome obesity will ultimately lead to behavioral changes sustaining weight loss [3], which may increase the risk of weight regains after the surgery. To date, the most effective standard obesity treatment is weight-loss lifestyle modification based on a combination of behavioral and cognitive approaches and nutrition and physical education.

Clinical psychological treatment approaches are pivotal and involve engaging patients in lifestyle modification and motivating them to successfully lose weight with the help of a multidisciplinary team [4]. Cognitive behavioral therapy (CBT) for obesity is aimed at not only losing weight but also preventing weight regain, thereby avoiding the dissatisfactory long-term results of earlier behavioral treatments. It firmly distinguishes between weight loss and weight maintenance, allowing patients to practice effective weight-maintenance strategies (eg, avoiding unrealistic weight goals and addressing obstacles to weight maintenance) [5]. One study applied a 12-week CBT program for obese people, resulting in a 6% reduction in body fat relative to the control group [6]. Moreover, a 20-week CBT intervention involving a 10-week main program followed by a 10-week less-intensive care program significantly improved body composition and improved soft drink consumption habits compared to the control group [7].

Although cognitive behavioral programs involving weekly clinic visits are known to be the most effective treatments for obesity, they place high demands due to time, cost, distance, status of endorsement, and difficulties securing child care [8]. A previous study found that people would prefer cost-effective and time-saving methods to lose weight [9]. Researchers have thus explored alternative methods for carrying out weight-loss programs, such as television, computers, and smartphone apps, to meet individual needs and to make obesity treatment more accessible. Among these, self-monitoring via smartphone apps has shown the greatest potential to make diet tracking easier and engaging because of its convenience and accessibility [10]. Despite the use of smartphone apps for self-monitoring, a *law of attrition* in digital health interventions still holds, whereby users stop using technology-based components over time. Because the effectiveness of treatments via digital tools is closely associated with the user’s extent of engagement [11], a high attrition rate is a critical issue in the assessment of the efficacy of digital intervention programs. Therefore, based on behavioral modification principles, periodic prompts that encourage healthy behaviors are one method to remind and motivate people to change their health behaviors. A systematic review of the use of technology tools to send periodic notifications about users’ behavior changes found them to be more effective than nontechnological notifications or no notifications [12]. However, this review only focused on the effectiveness of digital interventions for behavior change as a whole and did not investigate how to enhance engagement with the intervention.

The goal of this study was to test a novel approach to losing weight and maintaining the new weight after participation in an intensive and comprehensive human coaching program based on CBT modules via digital tools, such as the Noom Coach app and InBody Dial. The Noom Coach app is one of the most popular smartphone apps currently available; it has received higher quality assessment scores than other smartphone apps [13]. It allows participants to log their food intake, exercise activities, and weight, and to engage in in-app group activities, read in-app articles, and interact with a human coach via in-app messages. In-app group activity lets participants communicate with other participants and share their experience of healthy lifestyle trials. In-app articles deliver practical information about healthy lifestyles written by physicians, nutritionists, and clinical psychologists. In-app messages enable participants to receive individualized feedback from human coaches based on their own records presented on the Web-based dashboard. A Web-based dashboard is provided to the coaches to monitor participants’ data. InBody Dial is a body composition analyzer for the home linked to a mobile app, allowing users to conveniently measure their body composition. Furthermore, we addressed the self-sustainability of the promoted lifestyle change after the intervention. We hypothesized that individuals randomized to the digital CBT group would lose weight and better maintain their weight loss than individuals in the control group.

## Methods

### Participants

**Figure 1:**
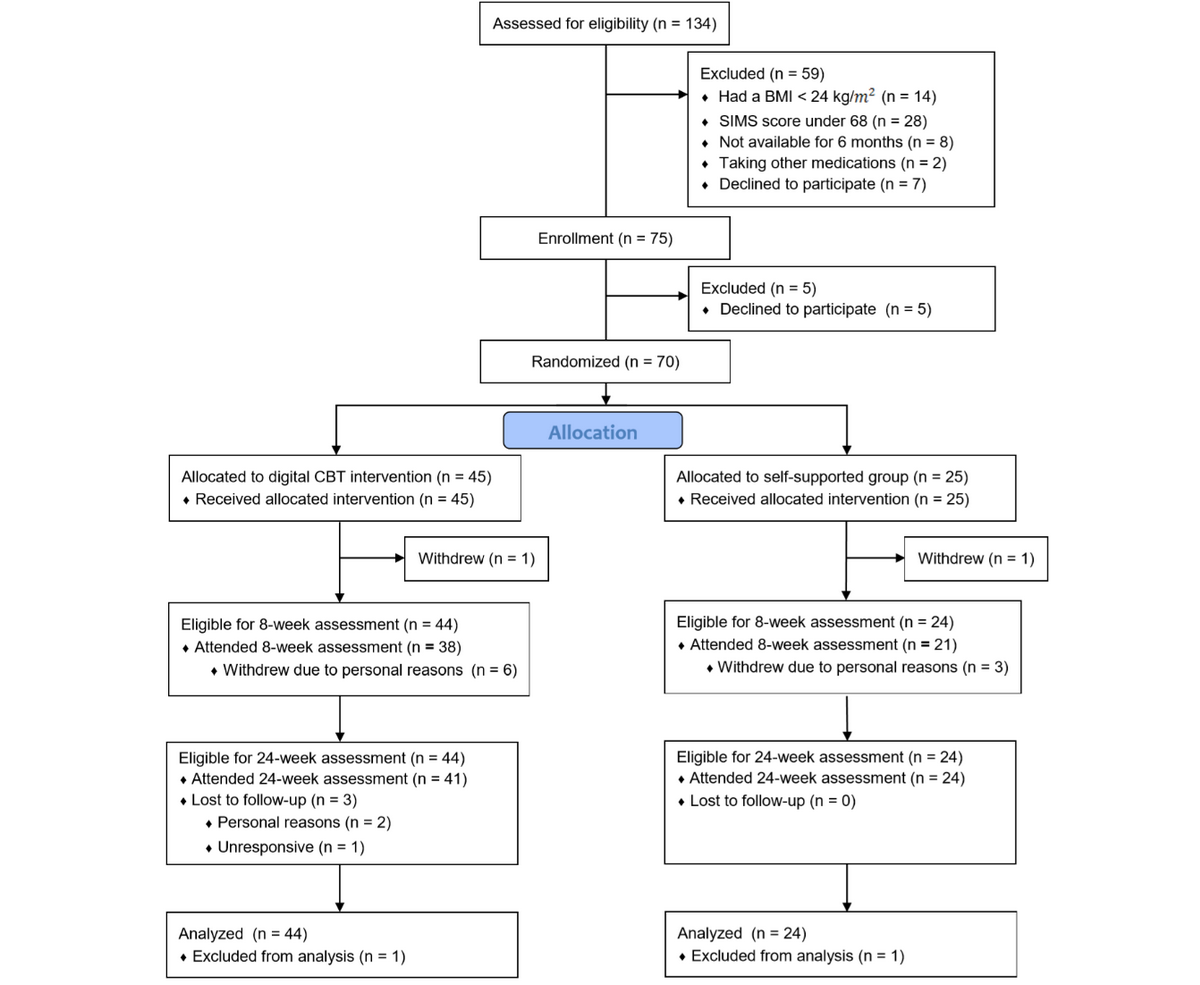
Digital cognitive behavioral therapy (CBT) CONSORT (Consolidated Standards of Reporting Trials) flow diagram. SIMS: Situational Motivation Scale.

A total of 70 female subjects were recruited between September and October 2017 through both online and offline boards of a university campus in Seoul, South Korea, and a social network service. Eligibility criteria included the following: 18-39 years of age, body mass index of 25-40 kg/m^2^, smartphone usage, and scores in the highest 40% (ie, scores above 68 out of 112 total) on the Situational Motivation Scale (SIMS). Participants were ineligible if they had a history of major medical problems, such as diabetes, angina, or stroke; a major psychiatric disorder involving hospitalization or medication in the past; and a current or planned pregnancy within the next 6 months. The flow of participants from recruitment to final assessment at 24 weeks is shown in Figure 1.

The Institutional Review Board of Seoul National University Hospital approved the study (approval number H-1707-122-872). All study participants provided written informed consent. This study was conducted to examine the clinical efficacy of the obesity digital CBT model and find factors predicting its efficacy. The study was registered with ClinicalTrials.gov (NCT03465306).

### Study Design

This was an open-label, active-comparator, randomized controlled trial (RCT). Following initial screening, all participants were asked to attend an orientation session where the study was described in more detail. Written informed consent and baseline measurements were obtained in person. Blood samples were taken in the morning after overnight fasting to avoid daily variations in activities. The basics of the tutorial and log-in procedures for both the Noom app and the InBody H20B (InBody Co) body composition analyzer were demonstrated to all participants during the orientation session of the study. The Noom app was mainly used to keep a food diary and deliver messages between the therapist and participants, while the InBody H20B analyzer was used to monitor and collect the body composition data of the participants. The randomization was designed to randomly assign 75 participants in total to a control (app only) group or a digital CBT (app + human CBT) group at a ratio of 1:2 in order to deliver a more powerful trial within resource constraints and to maximize the statistical power of predictor analysis (ie, within-group analysis) [14]. Randomization was performed by the project manager by drawing lots. The digital CBT group was given daily feedback and assignments from a psychologist, based on the CBT modules, for 8 weeks and could access the digital tools from the intervention period to the 24-week follow-up. The control group was instructed to use only a food diary without therapist intervention until the 24-week follow-up but was given the same digital tools and instruction as the digital CBT group. Thus, the control group underwent the same standard-of-care trial as the digital CBT group, except that it was asked to practice self-care. All participants were asked to visit at baseline, 8 weeks, and 24 weeks for objective measurements and completion of questionnaires, and they were each paid US $4 for attending each of the appointments. This study was conducted from September 2017 to April 2018.

### Assessment

The primary outcome was change in body weight. Other measures, such as change in BMI and body fat mass, were secondary outcomes. Anthropometric measurements were assessed by the InBody H20B analyzer at baseline, 8 weeks, and 24 weeks in light street clothing and without socks and shoes. For secondary outcomes, blood samples were collected at baseline and 8 weeks after a 10-hour fast. We examined serum insulin, leptin, glucose concentrations, aspartate aminotransferase, alanine aminotransferase, gamma-glutamyl transferase, total cholesterol, and triglyceride levels to assess the changes in these indices in relation to the change in body weight. The engagement criteria of the program were completing actions, such as responding to the daily assessment (responses per day), logging meals (meals per week), consuming green foods as defined by Noom [15] (logged per week), performing exercise (times per week), registering exercise time (minutes per week), recording steps taken (steps per week), logging weigh-ins (times per week), reading articles (articles per week), completing group posts (posts per week), posting group comments (comments per week), sending messages to the coach (messages per week), and liking group posts (likes per week). These criteria were used to assess the use of the app by each participant with objective measures.

Participants’ situational motivation toward the weight-loss program was assessed using an adapted version of the SIMS. The SIMS typically measures four types of motivation—intrinsic motivation, identified regulation, external regulation, and amotivation—to engage in a task (ie, the weight-loss program) at a specific point in time, with four items per subscale. The SIMS has demonstrated acceptable levels of reliability and validity in past research. The Body Shape Questionnaire-8C (BSQ-8C) is a brief version of the Body Shape Questionnaire (BSQ) consisting of eight items extracted from the full version measuring the extent of psychopathology of concerns about body shape. Higher values on the BSQ indicated more body dissatisfaction. Depression was assessed using the Korean version of the Beck Depression Inventory-II (K-BDI-II) scoring system. A total score of 0-9 indicated no depression, 10-15 indicated mild depression, 16-23 indicated moderate depression, and 24-63 indicated severe depression. Anxiety was measured using the 20-item Trait Anxiety Inventory (TAI) of the State-Trait Anxiety Inventory, with higher scores indicating greater trait anxiety. The Rosenberg Self-Esteem Scale (RSES) measure of self-esteem was used in this research with a 10-item scale consisting entirely of negatively worded items. Thus, higher scores implied lower self-esteem. Eating behavior notions were measured with the Dutch Eating Behavior Questionnaire (DEBQ), which identifies three distinct psychologically based eating behaviors: restrained eating, emotional eating, and external eating. It contains 33 items, with higher scores indicating a greater tendency to present subscale behavior. The frequency of occurrence of automatic negative thoughts associated with depression was assessed by the Automatic Thoughts Questionnaire (ATQ-30). The scores ranged from 30 to 150, where higher scores indicated more frequent automatic negative thoughts. All the psychological questionnaires were in Korean.

### Interventions

The intervention of this study was a multifactorial, daily-based personalized coaching program implemented by a psychologist using CBT modules via the digital platform. The digital CBT contents were based on programs proposed to clinicians [16] as a guide. We monitored and assessed various factors related to the behavior, cognition, mood, and motivation of each participant assigned to the digital CBT group.

The following were assessed every day using responses to questions and scores from the questionnaires: eating behaviors (eg, Where did you eat? What type of food did you have? How fast did you eat? and What time did you eat?), automatic thoughts (eg, What came to your mind when you were eating or thinking of food?), mood (eg, Score your mood from 0 to 100 regarding each type of negative mood: irritated, lonely, anxious, bored, and depressed), and motivation (eg, Score your status from 0 to 10 based on the following items: willingness to lose weight, importance of losing weight, assurance of losing weight, and helpfulness of this program to lose weight). Scores were used to individually track the daily patterns of the four factors—eating behaviors, automatic thoughts, mood, and motivation—and provide individualized interventions. As such, participants in the digital CBT group received daily self-report assessments in a Google survey form via text message on their phone. Participants were also instructed to log their dietary intake and physical exercise on a daily basis. Additionally, they were asked to measure their weight, BMI, and fat mass twice a week with the InBody H20B analyzer as soon as they woke up in the morning and were instructed to log their meals and physical activity by self-report on the Noom Coach app on a weekly basis.

After participants’ responses to the components related to the four factors were collected, digital mobile tools collected the data to allow the therapist to securely monitor participants’ progress through a Web-based dashboard. The participants received at least three individual messages from the coach every day, except on weekends and holidays, via the Noom Coach app. Furthermore, the therapist individually sent a daily report, a weekly report, and a midweek report (ie, Week 4) to the participants for the purpose of goal setting and to strengthen their motivation. Weekly group missions were provided to the digital CBT group based on the expectation that social supports (eg, communicating needs and building positive support) would intensify the motivation. When the participants were inactive for more than 3 consecutive days or asked for thorough counseling, the therapist phoned them and conducted motivational interviews. The motivational interviews could be implemented only once a week per person. The duration of the phone call did not exceed 15 minutes.

**Figure 2:**
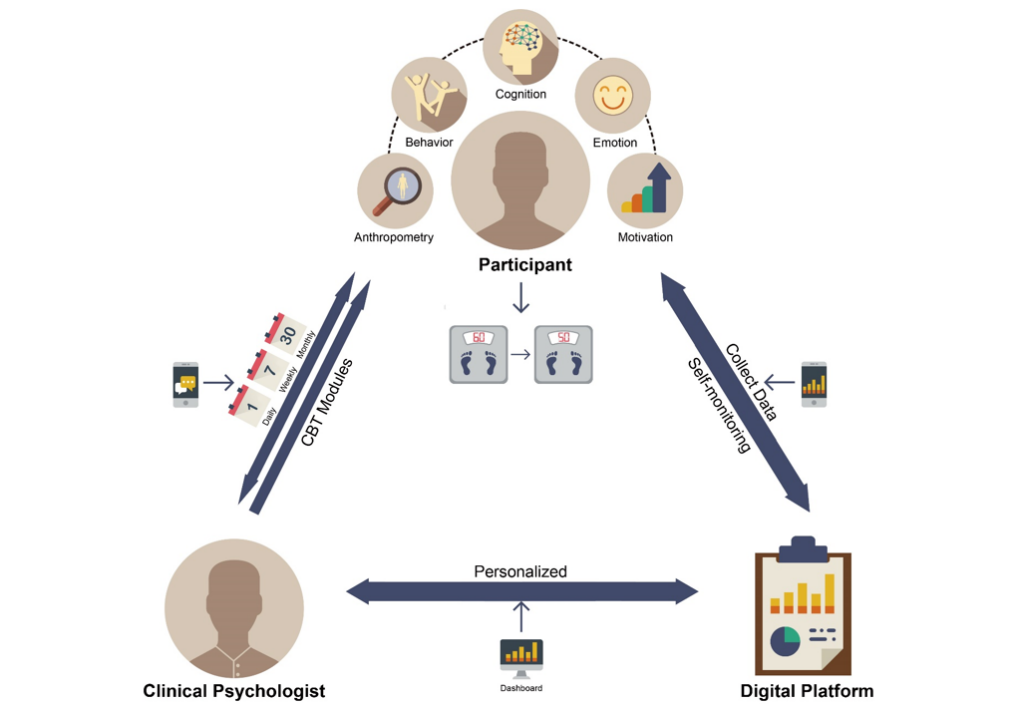
Diagram of the digital cognitive behavioral therapy (CBT) process.

**Figure 3:**
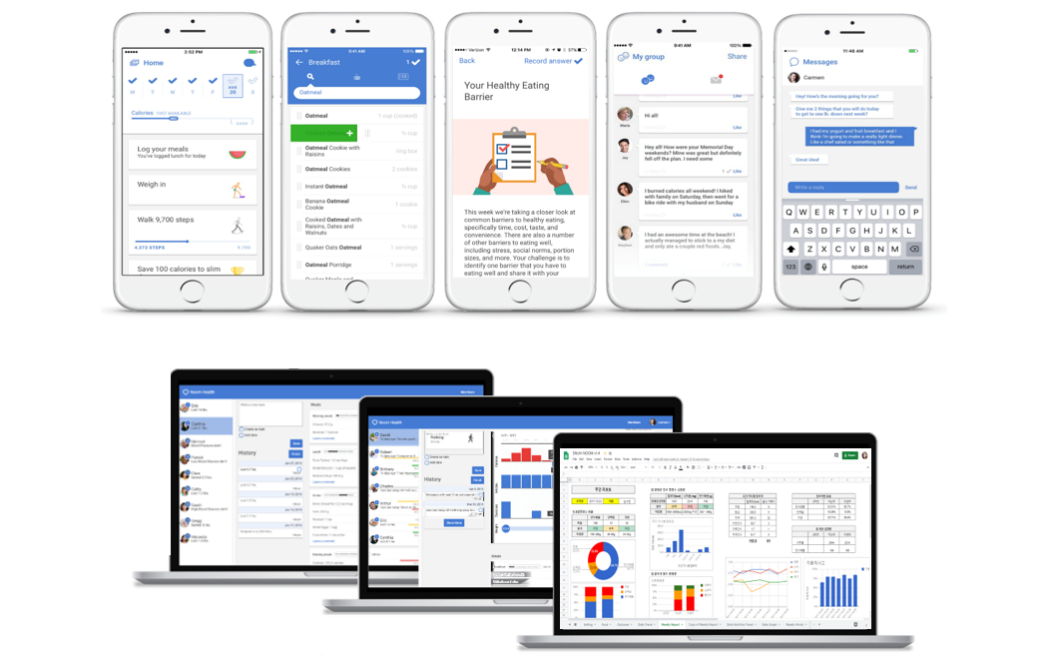
Screenshots of the digital platform (ie, mobile apps) for the participants (top) and screenshots of the digital platform (ie, dashboard) for the therapist (ie, clinical psychologist) (bottom).

All contents of the coaching messages, group missions, and articles were managed by a supervisor of the digital health care coach, who has a master-level degree in clinical psychology. She has trained as a behavioral therapist using CBT modules, such as self-monitoring, goal setting, problem solving, nutritional and physical activity education, stimulus control, challenging automatic thoughts, thought restructuring, and relapse prevention. Throughout the intervention, we expected the participants in the digital CBT group to experience a lifestyle change by finding a healthy pattern of living that fit each participant’s context. The diagram of the digital CBT process and features of the digital platform are presented in Figure 2 and Figure 3, respectively.

### Statistical Analysis

The sample size was selected to provide the study with a statistical power of 80% to detect clinically meaningful mean differences in weight loss of 5 kg with an SD of 7 kg in treatment effect, based on previous studies [17]. Assuming an average attrition rate of 10%, a sample of at least 70 subjects was selected. For differences in baseline characteristics, independent-sample *t* tests were used for continuous variables and a chi-square test of independence was used for categorical data assessing the demographic patterns of subjects.

We conducted the analysis following per-protocol principles. The participants who attended at either 8 or 24 weeks were included in the analysis of the applicable period without missing imputations. There were no outliers in the dataset. To investigate differences in the outcomes between the two groups, changes in the outcomes of weight, BMI, and fat mass were analyzed using an independent-sample *t* test. To investigate statistical differences between baseline and postintervention within a group, a paired *t* test was used. To detect statistical differences of the proportion within the thresholds and engagement rates between groups, a chi-square test was used. Correlation analysis using the Pearson correlation coefficient was used to investigate which variables at the baseline had a predictive role in changes in anthropometrics at 8 and 24 weeks. Receiver operating characteristic (ROC) curve analysis was undertaken to identify the optimum trade-off between sensitivity and specificity for cutoffs in weight-change distribution. For the ROC analysis in this study, we set a cutoff of 3% loss of initial body weight as a *good response* at 24 weeks for the digital CBT group data. The Youden index was used for the optimal cutoff. The results regarding the proportion of people who reached 5% weight-loss threshold are also reported to permit comparison with other previous studies. All analyses were conducted using SPSS Statistics for Windows, version 20 (IBM Corp), and statistical significance of two-tailed *P* values were set at .05. For multiple comparison correction, a threshold of *P*<.001 was used (ie, the *P* value threshold of .05 divided by 42, corresponding to two different time periods and 21 phenotypes).

## Results

### Overview

There were no significant differences between the randomization groups on key demographic characteristics (see Table 1). However, the DEBQ emotional eating scale (DEBQ-EM) (*P*=.001) and the DEBQ external eating scale (DEBQ-EX) (*P*=.049) scores of the two groups did differ at baseline. These differences between the groups were found after lots were drawn for the randomized control procedure. Participants had a mean age of 21.8 years (SD 3.3) and a mean BMI of 28.0 kg/m^2^ (SD 3.2).

**Table 1:** Baseline characteristics of participants in both groups.

Characteristic		Control (ie, app only) (n=25)	Digital CBT^a^ (ie, app + human CBT) (n=45)
Age (years), mean (SD)		21.0 (2.7)	22.3 (3.5)
**Anthropometric measures, mean (SD)**			
	Weight (kg)	71.9 (7.7)	74.5 (9.0)
	BMI (kg/m^2^)	27.7 (2.9)	28.2 (3.4)
	Fat mass (kg)	29.3 (6.0)	30.2 (6.8)
	Fat percent (%)	40.5 (4.8)	40.4 (5.4)
	Lean body mass (kg)	23.8 (3.3)	24.0 (2.6)
**Blood measures, mean (SD)**			
	Fasting glucose (mg/dL)	87.0 (8.1)	87.3 (7.4)
	Triglyceride (mg/dL)	92.2 (35.9)	93.2 (42.6)
	Total cholesterol (mg/dL)	184.7 (24.9)	191.1 (30.4)
	Alanine aminotransferase (U/L)	12.7 (6.9)	15.3 (11.9)
	Aspartate aminotransferase (U/L)	17.0 (4.7)	16.9 (4.8)
	Gamma-glutamyl transpeptidase (U/L)	15.3 (8.5)	21.3 (32.8)
	Leptin (ng/mL)	37.5 (14.7)	42.5 (15.3)
	Fasting insulin (µU/mL)	12.6 (6.1)	16.1 (9.1)
Homeostasis Model for Assessment of Insulin Resistance^b^, mean (SD)		2.8 (1.5)	3.5 (2.1)
**Scale or questionnaire (score), mean (SD)**			
	Situational Motivation Scale	77.0 (5.8)	76.1 (5.7)
	Body Shape Questionnaire-8C	34.8 (8.9)	36.2 (7.5)
	Beck Depression Inventory-II in Korean	14.7 (9.6)	13.6 (9.0)
	Trait Anxiety Inventory	47.8 (11.0)	48.0 (10.4)
	Rosenberg Self-Esteem Scale	21.9 (6.4)	19.8 (5.6)
	DEBQ^c^ restrained eating scale	30.6 (7.3)	29.9 (6.6)
	DEBQ emotional eating scale^d^	29.1 (11.6)	38.0 (10.1)
	DEBQ external eating scale^d^	32.0 (7.0)	34.9 (4.8)
	Automatic Thoughts Questionnaire	57.6 (26.0)	57.2 (22.3)
	Yale Food Addiction Scale	2.2 (1.7)	3.0 (1.7)
**Residence status, n (%)**			
	Living with family	10 (40)	27 (60)
	Living alone	8 (32)	8 (18)
	Living with roommates	7 (28)	9 (20)
	Others	0 (0)	1 (2)
**Number of attempts to lose weight by different methods, n (%)**			
	None	0 (0)	1 (2)
	Once	3 (12)	4 (9)
	Twice	12 (48)	15 (33)
	Three times	3 (12)	13 (29)
	Four times	4 (16)	8 (18)
	Five times	2 (8)	4 (9)
	Six times	1 (4)	0 (0)

^a^CBT: cognitive behavioral therapy.

^b^Insulin resistance = (insulin [µU/mL] × glucose [mg/dL]) / 405.

^c^DEBQ: Dutch Eating Behavior Questionnaire.

^d^There was a statistical difference between the two groups at baseline.

### Primary Outcome of Weight Change and Anthropometric Outcomes

**Figure 4:**
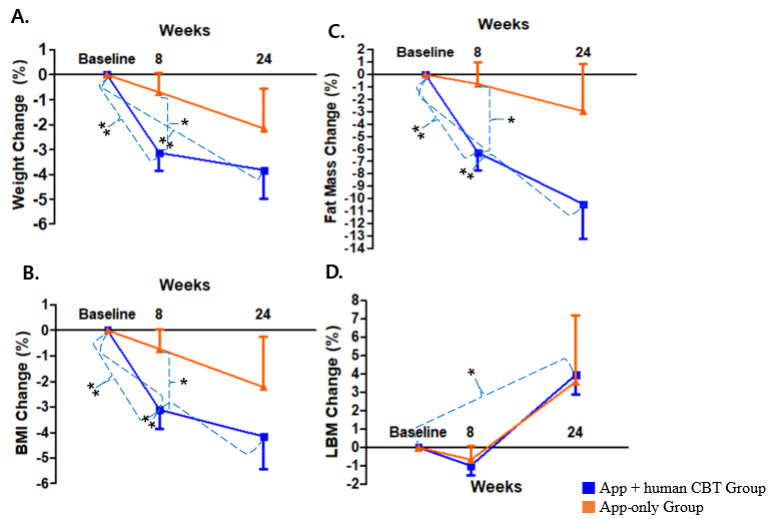
Patterns of changes in mean body weight (A), BMI (B), body fat mass (C), and lean body mass (LBM) (D). CBT: cognitive behavioral therapy. *P<.05; **P<.01.

**Figure 5:**
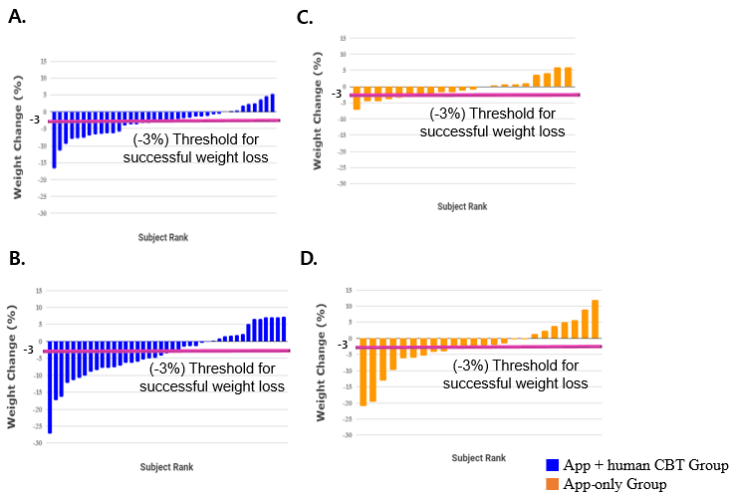
Weight change based on individual data from the experimental group at the 8-week follow-up (A), from the experimental group at the 24-week follow-up (B), from the control group at the 8-week follow-up (C), and from the control group at the 24-week follow-up (D). CBT: cognitive behavioral therapy.

The primary outcome (ie, weight change) was assessed at two time points—immediately after lifestyle change with digital CBT (8 weeks) and at the long-term follow-up without digital CBT (24 weeks)—to investigate the self-sustaining effect of lifestyle change induced by 8 weeks of digital CBT. Of the 70 randomized participants, 65 (93%) were assessed for the primary outcome—body weight—at 24 weeks and 5 (7%) were lost to follow-up. Figures 4 and 5 represents the mean weight change along with other anthropometric measures—BMI, body fat mass, and body lean mass—at each study time point. Participants in the digital CBT group showed significant changes in mean body weight at 8 weeks compared to the control group (–3.1%, SD 4.5, vs –0.7%, SD 3.4, *P*=.04) but not at 24 weeks. The proportion of subjects who showed *good response* was 45% (17/38) in the digital CBT group and 29% (6/21) in the control group at 8 weeks (*P*=.22), while at 24 weeks it was 54% (22/41) in the digital CBT group and 42% (10/24) in the control group (*P*=.35). In addition, the number reaching the conventional 5% weight loss from the baseline in the digital CBT group was significantly higher than in the control group at 8 weeks (12/38, 32%, vs 1/21, 4%, *P*=.02) but not at 24 weeks (18/41, 44%, vs 7/24, 29%, *P*=.24). Changes in mean BMI (–3.1%, SD 4.6, vs –0.7%, SD 3.5, *P*=.04) and body fat mass (–6.3%, SD 8.8, vs –0.8%, SD 8.1, *P*=.02) of the digital CBT group were also significant compared to the control group at 8 weeks but not at 24 weeks (see Multimedia Appendix 1, Table MA1-1). Body lean mass did not significantly differ between the two groups at both 8 and 24 weeks. Examining within-group changes, only the digital CBT group achieved significant weight changes, as well as BMI and body fat mass, at both 8 and 24 weeks; the digital CBT group achieved significant changes in lean body mass at 24 weeks but not at 8 weeks (see Multimedia Appendix 1, Tables MA1-2 and MA1-3).

**Figure 6:**
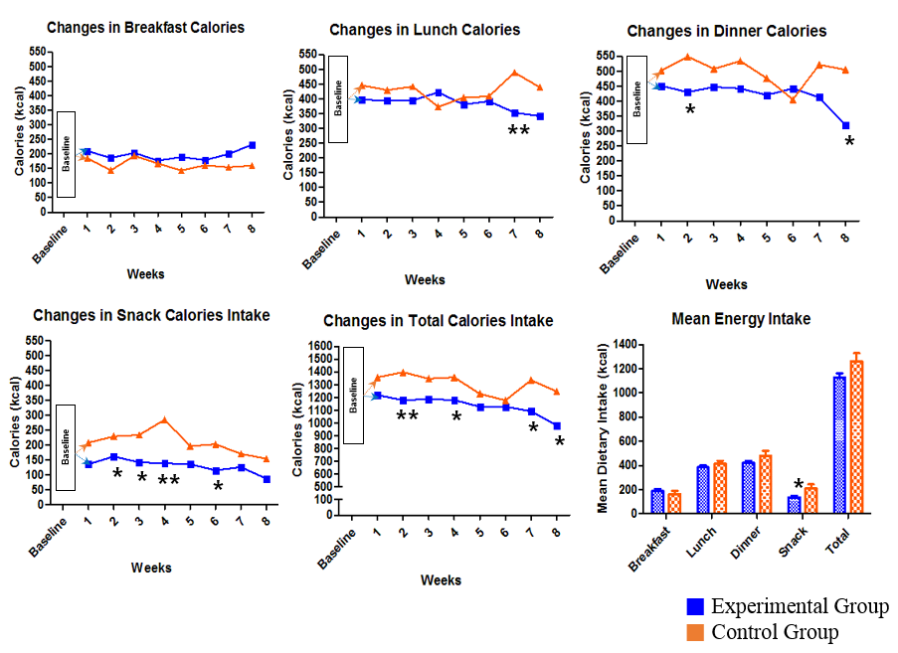
Changes in meal calories between experimental and control groups during the intervention period, as well as the contrast of mean energy intake between groups. *P<.05; ** *P*<.01.

### Secondary Outcomes: Metabolic and Psychological Outcomes

Multimedia Appendix 1, Table MA1-4, shows a comparison of the metabolic outcomes from baseline to 8 weeks in each group and by intervention condition. The mean decreases in leptin (–15.8%, SD 29.9, vs 7.2%, SD 35.9, *P*=.01), insulin (–4.4%, SD 35.2, vs 15.4%, SD 35.1, *P*=.048), and Homeostatic Model Assessment for Insulin Resistance (HOMA-IR) (–7.1%, SD 35.1, vs 14.4%, SD 41.2, *P*=.04) were significantly greater in the digital CBT group than in the control group. For within-group analysis, the changes in glucose (–2.91%, *P*=.04) and leptin (–15.82%, *P*=.003) of the digital CBT group were significant. No significant outcome changes were found in the control group. The mean percentage changes in psychological outcomes are shown in Multimedia Appendix 1, Table MA1-5, by intervention condition. There was no significant difference between the groups regarding the number of changes in psychological outcomes except for the change in the DEBQ-EM from baseline to 8 weeks (*P*=.048). Paired *t* test analysis showed significant changes in the BSQ-8C and DEBQ-EX scores at 8 and 24 weeks in both groups. However, the changes in the scores of the DEBQ restrained eating scale (DEBQ-RE) (*P*<.001) at 8 weeks, and those of the K-BDI-II (*P*=.001), TAI (*P*=.04), RSES (*P*=.03), and ATQ-30 (*P*=.02) at 24 weeks, appeared to be significant only in the digital CBT group (see Multimedia Appendix 1, Tables MA1-6 and MA1-7). Behavioral outcomes, measured via the Noom app, are represented as the amount of calorie intake and the pattern of weekly changes between the groups and the average energy intake of each group, as presented in Figure 3. Mean snack calories (*P*=.02) significantly differed between the two groups, and total calories (*P*=.06) had a tendency toward critical difference by intervention condition (see Figure 6 and Multimedia Appendix 1, Table MA1-8).

**Figure 7:**
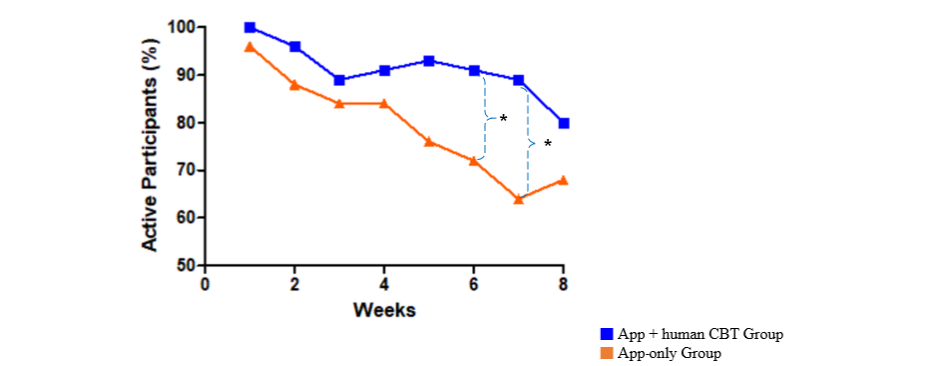
Patterns of changes in engagement rate of the experimental and control groups during the intervention period. *P<.05.

Lastly, the digital CBT group had a higher engagement rate when using digital tools than the control group, though it declined over time in both groups (see Figure 7 and Multimedia Appendix 1, Table MA1-9).

### Predictors of the Primary Outcome, Weight Change

#### Correlations Between the Primary Outcome and the Baseline Characteristics

**Figure 8:**
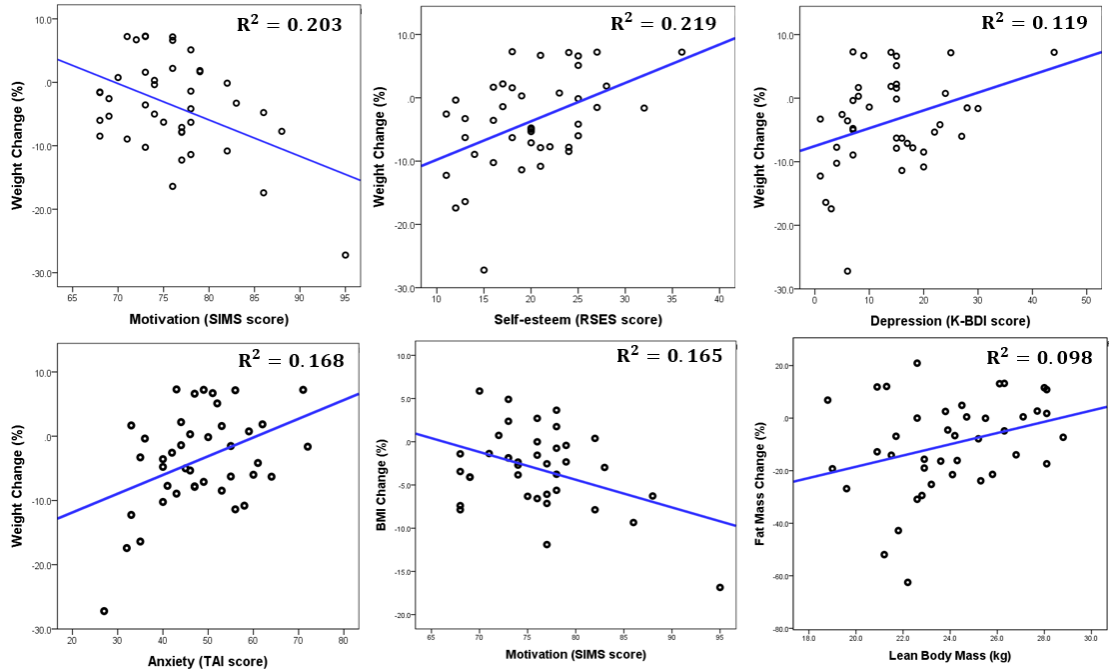
The correlation between weight change at the long-term follow-up period (24 weeks) and the level of motivation, self-esteem, depression, and anxiety at baseline. Also shown are the correlation between BMI change at the long-term follow-up and the level of motivation at baseline, and the correlation between fat mass change at the long-term follow-up and lean body mass at baseline. K-BDI: Korean version of the Beck Depression Inventory; RSES: Rosenberg Self-Esteem Scale; SIMS: Situational Motivation Scale; TAI: Trait Anxiety Inventory.

The baseline motivation, as measured by the SIMS, was significantly correlated with weight change at 8 weeks (*P*=.009) and 24 weeks (*P*=.003). Depression, as measured by the K-BDI-II (*P*=.03); anxiety, as measured by the TAI (*P*=.008); and self-esteem, as measured by the RSES (*P*=.002) at baseline also showed a significant correlation with weight change at 24 weeks but not at 8 weeks. Depression, anxiety, self-esteem, restrained eating behavior, external eating behavior, and automatic thoughts at baseline were significantly correlated with BMI change at 24 weeks. Lastly, lean body mass, anxiety, and self-esteem at baseline were significantly correlated with change in body fat mass at 24 weeks. Figure 8 illustrates the significant correlations between the predictive markers and the change of the anthropometric measures at 24 weeks. All the results of the correlation analysis are presented in detail in Multimedia Appendix 1, Table MA1-10. Multimedia Appendix 1, Figure MA1-1, also illustrates the correlations between predictive markers and the change of BMI.

#### Receiver Operating Characteristic Analysis Determining the Optimal Cutoff Scores of the Predictive Markers of Success in Weight Loss by Digital Cognitive Behavioral Therapy

Multimedia Appendix 1, Table MA1-11, shows the sensitivity and specificity of the baseline psychological characteristics showing significant correlations with weight change, the primary outcome. The definition of optimal statistical prediction threshold is weight loss of more than 3% of the initial body weight. This is an important threshold because our treatment was CBT as a lifestyle modification without any biological intervention. Both motivation and self-esteem had the greatest area under the curve (AUC) (0.63). The AUCs of depression and anxiety were 0.61 and 0.62, respectively. To predict a good response, the cutoff for motivation (SIMS score=76.5) provided a good trade-off between sensitivity (59%) and specificity (74%). Additionally, the cutoff for depression (K-BDI-II score=7.5), anxiety (TAI score=41.5), and self-esteem (RSES score=24.5) provided optimal sensitivity and specificity to predict a good response. Overall, motivation showed the best predictive performance.

#### Clinical Efficacy of Digital Cognitive Behavioral Therapy Based on the Optimal Cutoff Scores of the Predictive Markers in the Clinical Setting

**Figure 9:**
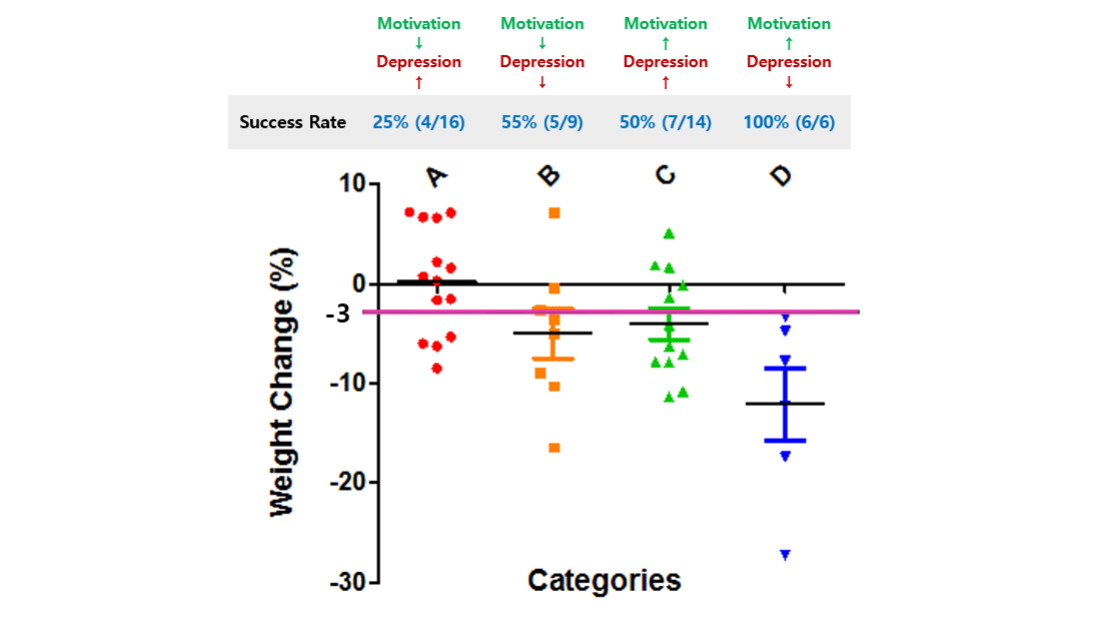
The clinical efficacy of digital cognitive behavioral therapy (CBT) by applying the optimal cutoff scores of the predictive markers in the clinical setting. The pink line represents the threshold for successful weight loss.

The high-motivation subgroup (SIMS scores >76.5) showed a 65% (13/20) probability of successful 3% weight loss, whereas the low-motivation subgroup (SIMS scores <76.5) showed a 36% (9/25) probability of successful 3% weight loss. Optimal predictive performance was achieved by combining both motivation and depression scores. The high-motivation plus low-depression subgroup (SIMS scores >76.5 and K-BDI-II scores <7.5) showed a 100% (6/6) probability of successful 3% weight loss. Other subgroups showed a lower probability of successful 3% weight loss: 55% (5/9) of the low-motivation and low-depression subgroup, 50% (7/14) of the high-motivation and high-depression subgroup, and 25% (4/16) of the low-motivation and high-depression subgroup (see Figure 9).

Even when the strict statistical threshold for multiple comparison corrections was applied, changes in weight, BMI, and fat mass from baseline to 8 weeks in the digital CBT group were considered significant (*P*<.001). The changes in the scores of the DEBQ-RE from baseline to 8 weeks, and in the K-BDI-II and DEBQ-EX scores from baseline to 24 weeks, in the digital CBT group were also significant after multiple corrections (*P*<.001). Furthermore, the changes in the scores of the BSQ-8C from baseline to 8 weeks and 24 weeks in both the digital CBT and control groups were considered significant after multiple corrections (*P*<.001).

## Discussion

### Principal Findings

This study successfully examined the efficacy of a newly developed, multifactorial, and daily-based personalized CBT model conducted by a psychologist via a digital platform for managing body weight, BMI, and body fat mass and showed a legacy effect even after the intervention terminated. This was performed by comparing this group to the active comparators using only the app as the control group. Furthermore, this study successfully explored the predictors for the efficacy of digital CBT from the baseline characteristics and recommended them as precision medicine biomarkers, namely, depression, anxiety, self-esteem, and motivation.

Among mobile health (mHealth) RCTs for obesity, this study has unique implications regarding the application of CBT strategies by a human coach in the intervention. This study, therefore, contributes to the broader literature on weight-loss treatments that involve human factors. There have been widespread studies of mHealth approaches to weight-loss programs [18-33]. There are several studies of obesity treatments that did not investigate CBT settings; these include studies of human-based mHealth RCTs [18,21,24,29,34,35], human-based mHealth but without RCT design [17,26,30], and mHealth not based on human factors but with RCT design [19,20,22,25,27,28,32,33]. There are also several studies of human-based RCT designs, including CBT settings for obesity but not mHealth procedures (ie, telephone, website, face-to-face, and others) [16,36-40].

This study is comparable to other mHealth RCTs. The mean percentage weight loss of our study was 4% of initial body weight, and previous mHealth RCTs reported a mean percentage weight loss ranging from 1% to 3% [24,29,32,34]. Moreover, this study successfully showed weight maintenance. Most interventions for obesity have shown a tendency to regain weight after discontinuing the treatment [11,16,24,29,32,41-43], but our digital CBT intervention showed a sustained trend of further decrease even up to 16 weeks after cessation of the 8-week intervention. This affords solid support for the assumption that digital CBT promotes an overall healthy lifestyle. However, because we do not have data beyond a 1-year period, a direct comparison with previous studies is not feasible. Preventing weight regain at 24 weeks is closely related with a decrease in body fat mass and an increase in lean body mass at 8 weeks, which are relevant to physical activity rate and nutrition status [44,45]. Indeed, an improvement in both physical activity and diet, representing changes in lifestyle, leads to healthy body composition. Therefore, the patterns of changes in not only body weight but also body fat mass and lean body mass may imply that the participants in the digital CBT group experienced self-sustainable transitions in daily decision making for a healthy life.

With regard to the appropriate threshold, previous behavioral weight-loss studies often reported 5% weight loss in the majority of participants [16,26,30]. Conventionally, several studies adopted a 5% threshold as a clinically significant threshold [16,19,26,29]. However, in contrast to the conventional 5% threshold, we adopted a tempered 3% weight-loss threshold as the *good response* threshold for two main reasons. First, the duration of the active intervention period in this study was shorter than in other studies and only persisted transiently for the initial 2 months. The majority of previous behavioral studies had a full 6-month active intervention design [16,36,37]. However, the duration of the active intervention period in our study was only 8 weeks (2 months). There was no intervention delivered after 8 weeks (2 months) until the 6-month time point. Thus, the subjects did not receive the intervention during the remaining 4 months after the initial 2-month active intervention. Second, the components of the intervention in this study did not include extreme restrictions or requirements in either diet or exercise. The main goal of our intervention was to implement sustainable weight management skills by learning an appropriate behavioral process as well as establishing new cognitive processes. Therefore, the weight loss per se could be weaker than with the stringent diet restrictions and exercise requirements of a behavioral program during the intervention. In addition to the 3% threshold, we also reported the results based on the conventional 5% threshold to allow a direct comparison of clinical efficacy between studies.

Regarding personalization, our digital CBT was fully tailored to each participant’s characteristics in multifactorial domains: the behavioral, cognitive, emotional, motivational, and physical domains. The therapist in our study altered the feedback styles based on data from five types of domains for every participant and conducted intensive daily monitoring. Most of the previous RCTs on mHealth interventions for obesity—those not based on human factors—considered one or two factors of individual symptoms that led to the implementation of homogeneous interventions [18,19,22,31]. Although there are some interventions that use custom algorithms to provide individualized feedback, they only focus on diet, physical activity, weight loss, or any two of these [27,28,32,46]. Furthermore, some earlier mHealth RCTs for obesity based on human factors only dealt with diet and physical activities [21,24]. One study managed three domains for the intervention: diet, physical activities, and eating behaviors [29]. However, instructions on behavior change strategies were not delivered by smartphone but by attending weekly group sessions for the first phase of the intervention. The study was deficient in other principal factors, such as emotional, cognitive, and motivational domains, implying insufficient potentiality for long-term lifestyle change. Because cognitive conceptualization and emotional regulation process are naturally associated with behavioral patterns, consideration of all these components can allow changes in one’s lifestyle and ultimately solve problems related to obesity [47]. Therefore, it is important to address the respective multifactorial domains so as to conduct tailored treatment for individuals with fully integrated techniques. Our digital CBT strategies operate in a fully comprehensive system that deals with behavioral, cognitive, emotional, motivational, and physical factors and allows integrated mediation to successfully manage obesity.

After examining aspects of temporal strategies for intervention, we arranged three different time points (ie, daily, weekly, and monthly points) and initiated a daily human-agent intervention in an mHealth RCT for obesity. All previous face-to-face, electronic health (eHealth), and mHealth RCTs for obesity treatment have been either weekly- or monthly-based interventions delivered by therapists [7,16,21,24,25,29,36-38]. Temporal strategies can influence the engagement rate, which is closely related to treatment outcomes [48]. Unfortunately, according to a systematic review, mHealth RCTs related to weight-loss programs suffer from a high attrition rate of more than 30% [49]. Our digital CBT trial, however, showed high in-app activity rates as well as engagement in the intervention program. Only one participant in the digital CBT group had to withdraw for personal reasons; 80% of the participants were active until the end of the treatment session. One possible reason for these outcomes is that our digital CBT intervention effectively managed participants’ motivation to lose weight as well as participate in the program. Our intervention did this by delivering individualized messages every day, based on data from the in-app database and daily assessment of various psychological factors using CBT modules, as well as facilitating real-time access to the therapist. Additionally, the midreport, employed as a monthly intervention, allowed personalized precision treatment based on initial psychological conditions to keep participants motivated. Thus, the engagement rate of the digital CBT group improved from 91% to 93%, whereas the engagement rate in the control group dropped from 84% to 76% between Week 4 and Week 5.

Through our digital CBT, changes in biological indexes, leptin, insulin, and HOMA-IR indicated that factors related to physical health can be successfully improved. Moreover, we also successfully managed motivation, emotion, cognition, and behavior. The level of self-body-image satisfaction and external eating behaviors was improved in both groups. This indicates that simply including the standard mHealth treatment in the control group in our study was practical for improving body image perception and external eating habits. Digital CBT improved the level of depression, anxiety, self-esteem, and automatic thoughts related to depression. In fact, the DEBQ-EM and DEBQ-EX scores showed a significant difference between the two groups at baseline but were not notably correlated with the primary measures at baseline. This may be considered a random circumstance of randomization. Therefore, these differences can be interpreted as not affecting the main outcomes of our study. Furthermore, a significant difference in reported snack calorie intake between the two groups suggests that our digital CBT intervention had an impact on managing snack calories compared to other meals. Stress is highly correlated with the frequency of snacks [50]. Thus, it is possible that our digital CBT intervention affected snack calorie intake by finding individualized stress coping strategies, restructuring cognitive structures of automatic negative eating or weight-related thoughts, and developing regular and balanced eating behaviors. Therefore, this provides evidence that the participants in the digital CBT group changed their lifestyle to constantly manage their weight.

This study can be considered a practical one because it explored clinical markers that predict the effect of digital CBT and suggested plausible criteria that can be applied to clinical settings. The follow-up results at 24 weeks in this study showed that the levels of motivation, depression, anxiety, and self-esteem were the predictive markers of weight loss based on the digital CBT intervention. Some of our results regarding the predictors of weight control conflict with the findings of previous research [51], but they are consistent with recent findings that the level of motivation is the strongest predictive trait for weight control [52,53]. We defined people who lost less than 3% of their baseline weight as poor responders to the treatment. Thus, people with a SIMS score lower than 76.5 are recommended to find and pursue their own way of enhancing their motivation to lose weight before they undertake digital CBT. Furthermore, a person whose score is higher than 7.5 on the K-BDI-II, 41.5 on the TAI, or 24.5 on the RSES is encouraged to handle the relevant issue before, or at the same time as, digital CBT. This will prevent further distress from repeated failure to control weight, save limited resources, and allow better concentration in individuals with a higher chance of success in weight control.

Considering the comparator of this study as the best active comparator without human coaching, digital CBT is a competent intervention for obesity in the current situation in the digital health care industry. We provided education on how to log meals and exercise as well as how to use InBody Dial and the mobile app, not only to the digital CBT group but also to the control group during the orientation. Thus, the control group in this study can be defined as an active group as in previous studies [19,22,31]. As expected, the control group in this study showed favorable results. Therefore, the results of this study are superior and significant compared to those of previous studies of digital health care interventions.

### Limitations

While the results are highly promising, the study is not without limitations. First of all, the participants were limited to those in their 20s and 30s, resulting in limited generalizability. Second, since this is not a blinded study, an observer bias could have been generated. Thus, an implication of this study that should be noted is that it tested the digital CBT and did not validate it. Third, the sample size was relatively small (N=70). Therefore, most of the results did not pass the strict multiple-comparison-corrected *P* threshold. Fourth, the follow-up period needs to be extended to increase the reliability and validity of our results. Accordingly, we recommend that future studies examine more information on personal characteristics, such as single nucleotide polymorphisms (SNPs) and daily patterns of digital phenotypes for individuals within in-app data, in order to enhance the interpretation of the efficacy of digital-based interventions. Fifth, the total amount of food calories in the app might have been underestimated because the amount per serving for diverse types of food was not precise and people may have miscalculated their food intake. The primary reason for errors in food records is that most people have difficulties in estimating food portions [54]. The discrepancy in food choice between the food diary and actual meals (ie, recording similar but not exact menus, skipping reports of foods eaten, or logging foods not offered) could explain the remainder of the total miscalculation [55]. Thus, we suggest that a direct assessment of food choice and intake, such as buffet tests, should be performed in parallel with logging intake in the food diary on the app for future research. In addition, it should be noted that it is necessary to involve dietitians on multidisciplinary health care teams for obesity CBT, as their evaluations of dietary assessment and nutritional advice would greatly strengthen the efficacy of the intervention. Lastly, there is a feasibility issue regarding the digital CBT of this study since it is intensive and costly, requiring daily intervention by therapists trained in both physical and mental health care. Therefore, more research involving human factors in technology-based treatments should be conducted to collect enough data to create automatic functions, thereby decreasing the burdens of therapists in the future.

### Conclusions

For the first time, we discovered that human-based digital CBT is capable of treating obesity using digital tools. Anthropometric measures, such as body weight and body compositions, were comparably improved by the digital CBT model as well as physiological indices and obesity-related psychological factors. There was no relapse in weight change after the end of the intervention. We also found predictable psychological markers to estimate the efficacy of the digital CBT treatment for obesity. This will open up new aspects of digital precision remedies for obesity in the digital health care industry.

## Appendix

Multimedia Appendix 1 Changes in outcomes from baseline, correlations, and predicting efficacy of digital cognitive behavioral therapy.

Multimedia Appendix 2 CONSORT-eHEALTH checklist (V 1.6.1).

## Abbreviations

ATQ-30: Automatic Thoughts Questionnaire
AUC: area under the curve
BSQ: Body Shape Questionnaire
BSQ-8C: Body Shape Questionnaire-8C
CBT: cognitive behavioral therapy
DEBQ: Dutch Eating Behavior Questionnaire
DEBQ-EM: Dutch Eating Behavior Questionnaire emotional eating scale
DEBQ-EX: Dutch Eating Behavior Questionnaire external eating scale
DEBQ-RE: Dutch Eating Behavior Questionnaire restrained eating scale
eHealth: electronic health
HOMA-IR: Homeostatic Model for Assessment of Insulin Resistance
K-BDI-II: Korean version of the Beck Depression Inventory-II
mHealth: mobile health
NRF: National Research Foundation of Korea
RCT: randomized controlled trial
ROC: receiver operating characteristic
RSES: Rosenberg Self-Esteem Scale
SIMS: Situational Motivation Scale
SNP: single nucleotide polymorphism
SNU: Seoul National University
TAI: Trait Anxiety Inventory
