# Supplementary material for: Multidimensional Cognitive Behavioral Therapy for Obesity Applied by Psychologists Using a Digital Platform: Open-Label Randomized Controlled Trial
Source: JMIR Mhealth Uhealth. 2020 Apr 30;8(4):e14817. doi: 10.2196/14817 (PMC7226050; doi:10.2196/14817)
Supplement: Multimedia Appendix 1 [file mhealth_v8i4e14817_app1.docx]

Table MA1-1. Comparison of Main Outcomes from Baseline by Intervention Condition

| **Outcome** | **Nª** | **Control** | **Nªª** | **Digital CBT** | ***p* (2-tailed)** |
| --- | --- | --- | --- | --- | --- |
| **Weight Change, %, mean (SD)** |  |  |  |  |  |
| Baseline to 8 Weeks  Baseline to 24 Weeks | 21  24 | -0.7 (3.4)  -2.7 (7.8) | 38  41 | -3.1 (4.5)  -3.7 (7.5) | .036*  .620 |
| **BMI Change, %, mean (SD)** |  |  |  |  |  |
| Baseline to 8 Weeks  Baseline to 24 Weeks | 21  24 | -0.7 (3.5)  -3.1 (9.6) | 38  41 | -3.1 (4.6)  -4.29 (8.2) | .043*  .597 |
| **Fat Mass Change, %, mean (SD)** |  |  |  |  |  |
| Baseline to 8 Weeks  Baseline to 24 Weeks | 21  24 | -0.8 (8.1)  -3.8 (18.7) | 38  41 | -6.3 (8.8)  -9.9 (18.1) | .021*  .200 |
| **LBM Change, %, mean (SD)** |  |  |  |  |  |
| Baseline to 8 Weeks  Baseline to 24 Weeks | 21  24 | -0.7 (3.4)  0.4 (17.8) | 38  41 | -1.0 (3.3)  2.5 (6.8) | .721  .519 |

**ª**Number of participants in control group, **ªª**Number of participants in Digital CBT group *p < .05; BMI = Body mass index; LBM = Lean Body Mass; CBT = Cognitive Behavioral Therapy.

Table MA1-2. Changes in main outcomes from baseline to 8 weeks and 24 weeks in Digital CBT group

| **Outcome** | **Baseline (n=45)** | **8 weeks**  **(n=38)** | ***p* (2-tailed) ª** | **24 weeks (n=41)** | ***p* (2-tailed)ªª** |
| --- | --- | --- | --- | --- | --- |
| **Weight, kg, mean (SD)** | 74.5 (9) | 71.5 (9.4) | <.001§ | 71.8 (10.8) | 0.003** |
| **BMI, kg/**$\boldsymbol{m}^{\boldsymbol{2}}$**, mean (SD)** | 28.2 (3.4) | 27 (3.5) | <.001§ | 26.9 (4) | 0.002** |
| **Fat Mass, kg, mean (SD)** | 30 (6.1) | 28.1 (6.7) | <.001§ | 27.4 (8.3) | 0.001** |
| **LBM, kg, mean (SD)** | 24 (2.7) | 23.6 (2.7) | 0.065 | 24.6 (2.7) | 0.023* |

**ª**Change from baseline to 8 weeks, **ªª**Change from baseline to 24 weeks, *p < .05; **p < .01; §These were significant after further multiple comparison corrections; BMI = Body mass index; LBM = Lean body mass; CBT = Cognitive Behavioral Therapy.

Table MA1-3. Changes in main outcomes from baseline to 8 weeks and 24 weeks in control group

| **Outcome** | **Baseline (n=25)** | **8 weeks**  **(n=21)** | ***p* (2-tailed) ª** | **24 weeks (n=24)** | ***p* (2-tailed)ªª** |
| --- | --- | --- | --- | --- | --- |
| **Weight, kg, mean (SD)** | 71.9 (7.7) | 71.6 (7.8) | 0.312 | 70.3 (8.5) | 0.091 |
| **BMI, kg/**$\boldsymbol{m}^{\boldsymbol{2}}$**, mean (SD)** | 27.7 (2.9) | 27.5 (2.8) | 0.270 | 26.8 (2.1) | 0.109 |
| **Fat Mass, %, mean (SD)** | 29.5 (6.4) | 29 (5.5) | 0.373 | 28 (5.9) | 0.175 |
| **LBM, kg, mean (SD)** | 23.8 (3.3) | 23.2 (2.4) | 0.307 | 23.9 (5.4) | 0.987 |

**ª**Change from baseline to 8 weeks, **ªª**Change from baseline to 24 weeks; BMI = Body mass index; LBM = Lean body mass; CBT = Cognitive Behavioral Therapy.

Table MA1-4. Changes in metabolic outcomes from baseline to 8 weeks in each group and by intervention condition

|  | **Control** | | | **Digital CBT** | | | **Control vs. Digital CBT** |
| --- | --- | --- | --- | --- | --- | --- | --- |
| **Outcome** | **Baseline**  **(n=25)** | **8 weeks**  **(n=21)** | ***P* (2-tailed)** | **Baseline**  **(n=45)** | **8 weeks**  **(n=34)** | ***P* (2-tailed)** | ***p* (2-tailed)** |
| **Glucose, mg/dL, mean (SD)** | 87 (8.1) | 85 (6.4) | 0.130 | 87.3 (7.4) | 84.1 (8.7) | 0.038* | .786 |
| **Triglyceride, mg/dL, mean (SD)** | 92.2 (35.9) | 96.3 (46.2) | 0.783 | 93.2 (42.6) | 93.3 (37.5) | 0.815 | .704 |
| **Total Cholesterol, mg/dL, mean (SD)** | 184.7 (24.9) | 189.1 (27.6) | 0.146 | 191.1 (30.4) | 191.2 (30.4) | 0.477 | .394 |
| **ALT, U/L, mean (SD)** | 12.7 (6.9) | 11.3 (4) | 0.439 | 15.3 (11.9) | 16 (16) | 0.826 | .973 |
| **AST, U/L, mean (SD)** | 17 (4.7) | 15.9 (2.6) | 0.556 | 16.9 (4.8) | 19.6 (14.6) | 0.345 | .392 |
| **GGT, U/L, mean (SD)** | 15.3 (8.5) | 13.8 (3.8) | 0.527 | 21.3 (32.8) | 16.8 (14.7) | 0.148 | .139 |
| **Leptin, ng/ml, mean (SD)** | 37.5 (14.7) | 38.5 (17.8) | 0.722 | 42.5 (15.3) | 34.1 (17.7) | 0.003** | .013* |
| **Insulin, µU/mL, mean (SD)** | 12.6 (6.1) | 13.8 (6.3) | 0.185 | 16.1 (9.1) | 14.4 (9.4) | 0.338 | .048* |
| **HOMA-IR** | 2.8 (1.5) | 2.9 (1.5) | 0.517 | 3.4 (1.8) | 3 (1.9) | 0.191 | .044* |

*p < .05; **p < .01; ALT = Alanine Aminotransferase; AST = Aspartate Aminotransferase; GGT = Gamma-Glutamyl Transpeptidase; HOMA-IR = Homeostasis model for assessment of insulin resistance; CBT = Cognitive Behavioral Therapy.

Table MA1-5. Comparison of Psychological Outcomes from Baseline by Intervention Condition

| **Outcome** | **Nª** | **Control** | **Nªª** | **Digital CBT** | ***p* (2-tailed)** |
| --- | --- | --- | --- | --- | --- |
| **BSQ-8C Change, %, mean (SD)** |  |  |  |  |  |
| Baseline to 8 Weeks  Baseline to 24 Weeks | 21  24 | -6.6 (13.2)  -14.9 (15.9) | 36  41 | -10.8 (14.8)  -8.8 (14.8) | .287  .122 |
| **K-BDI-II Change, %, mean (SD)** |  |  |  |  |  |
| Baseline to 8 Weeks  Baseline to 24 Weeks | 21  24 | 1.7 (12.3)  -0.2 (20.1) | 36  41 | -1 (13.9)  -7.4 (13.6) | .460  .090* |
| **TAI Change, %, mean (SD)** |  |  |  |  |  |
| Baseline to 8 Weeks  Baseline to 24 Weeks | 21  24 | 0.5 (9.2)  -0.1 (15.1) | 36  41 | -2.4 (15.5)  -4.7 (14.3) | .451  .221 |
| **RSES Change, %, mean (SD)** |  |  |  |  |  |
| Baseline to 8 Weeks  Baseline to 24 Weeks | 21  24 | 0.2 (9.9)  -2.9 (22.9) | 36  41 | 0.4 (12.2)  -3.5 (10.1) | .947  .890 |
| **DEBQ-RE Change, %, mean (SD)** |  |  |  |  |  |
| Baseline to 8 Weeks  Baseline to 24 Weeks | 21  24 | 14.9 (30)  4.7 (16.4) | 36  41 | 23.2 (38.8)  3.5 (15.5) | .405  .373 |
| **DEBQ-EM Change, %, mean (SD)** |  |  |  |  |  |
| Baseline to 8 Weeks  Baseline to 24 Weeks | 21  24 | 21.6 (56.9)  2.2 (32.6) | 36  41 | -2.8 (34.4)  -9 (31.1) | .048**  .175 |
| **DEBQ-EX Change, %, mean (SD)** |  |  |  |  |  |
| Baseline to 8 Weeks  Baseline to 24 Weeks | 21  24 | -6.2 (14.7)  -10.6 (22.1) | 36  41 | -9.1 (17.2)  -13.6 (17.8) | .523  .556 |
| **ATQ-30 Change, %, mean (SD)** |  |  |  |  |  |
| Baseline to 8 Weeks  Baseline to 24 Weeks | 21  24 | 0.7 (13)  -4.9 (25.3) | 36  41 | 1.1 (17.7)  -5.9 (15.9) | .920  .850 |
| **YFAS Change, %, mean (SD)** |  |  |  |  |  |
| Baseline to 8 Weeks  Baseline to 24 Weeks | 21  24 | 1.4 (34.7)  1.8 (37.7) | 36  41 | 9.5 (33.8)  -1.4 (26.9) | .388  .694 |

**ª**Number of participants in control group, **ªª**Number of participants in Digital CBT group, *p < .1, **p < .05; CBT = Cognitive Behavioral Therapy; BSQ-8C = Body Shape Questionnaire; K-BDI-II = Beck Depression Inventory-II in Korean; TAI = Trait-Anxiety Inventory; RSES = Rosenberg Self Esteem Scale; DEBQ-RE = Dutch Eating Behavior Questionnaire-Restrained; DEBQ-EM = Dutch Eating Behavior Questionnaire-Emotional; DEBQ-EX = Dutch Eating Behavior Questionnaire-External; ATQ-30 = Automatic Thoughts Questionnaire; YFAS = Yale Food Addiction Scale.

Table MA1-6. Changes in psychological outcomes from baseline to 8 weeks and 24 weeks in Digital CBT group

| **Outcome** | **Baseline (n=45)** | **8 weeks**  **(n=36)** | ***p* (2-tailed)ª** | **24 weeks (n=41)** | ***p* (2-tailed)ªª** |
| --- | --- | --- | --- | --- | --- |
| **BSQ-8C, score, mean (SD)** | 36.2 (7.5) | 30.5 (8.4) | <.001§ | 31.7 (9.5) | <.001§ |
| **K-BDI-II, score, mean (SD)** | 13.6 (9) | 12.3 (8.2) | .677 | 9.1 (6) | .001§ |
| **TAI, score, mean (SD)** | 48 (10.4) | 46.3 (10.9) | .368 | 45.3 (9.4) | .041* |
| **RSES, score, mean (SD)** | 19.8 (5.6) | 19.6 (6) | .857 | 19 (5.6) | .033* |
| **DEBQ-Restrained, score, mean (SD)** | 29.9 (6.6) | 35.5 (9) | <.001§ | 31.7 (8.7) | .154 |
| **DEBQ-Emotional, score, mean (SD)** | 38 (10.1) | 35.1 (12.2) | .288 | 34.3 (11.2) | .072 |
| **DEBQ-External, score, mean (SD)** | 34.9 (4.8) | 31.6 (7) | .003** | 29.2 (7.7) | <.001§ |
| **ATQ-30, score, mean (SD)** | 57.2 (22.3) | 57.5 (23.6) | .709 | 50.4 (20.5) | .023* |
| **YFAS, score, mean (SD)** | 3 (1.7) | 3.3 (1.8) | .100 | 2.7 (1.6) | .742 |

**ª**Change from baseline to 8 weeks, **ªª**Change from baseline to 24 weeks, *p < .05; **p < .01; §These were significant after further multiple comparison corrections; CBT = Cognitive Behavioral Therapy; BSQ-8C = Body Shape Questionnaire; K-BDI-II = Beck Depression Inventory-II in Korean; TAI = Trait-Anxiety Inventory; RSES = Rosenberg Self Esteem Scale; DEBQ = Dutch Eating Behavior Questionnaire; ATQ-30 = Automatic Thoughts Questionnaire; YFAS = Yale Food Addiction Scale.

Table MA1-7. Changes in psychological outcomes from baseline to 8 weeks and 24 weeks in control group

| **Outcome** | **Baseline (n=25)** | **8 weeks**  **(n=21)** | ***p* (2-tailed)†** | **24 weeks (n=24)** | ***p* (2-tailed)‡** |
| --- | --- | --- | --- | --- | --- |
| **BSQ-8C, score, mean (SD)** | 34.5 (8.9) | 31.9 (9.9) | .033* | 27.5 (10.2) | <.001§ |
| **K-BDI-II, score, mean (SD)** | 14.7 (9.6) | 17 (10.8) | .525 | 14.6 (12.2) | .962 |
| **TAI, score, mean (SD)** | 47.8 (11) | 48.8 (12.4) | .815 | 47.8 (10.5) | .982 |
| **RSES, score, mean (SD)** | 21.9 (6.4) | 22.3 (5.9) | .942 | 20.9 (6.2) | .539 |
| **DEBQ-Restrained, score, mean (SD)** | 30.6 (7.3) | 32.9 (5.1) | .066 | 33 (7.9) | .172 |
| **DEBQ-Emotional, score, mean (SD)** | 29.1 (11.6) | 32.9 (11) | .268 | 30.4 (11.7) | .745 |
| **DEBQ-External, score, mean (SD)** | 32 (7) | 30.4 (5.4) | .042* | 28.2 (7.8) | .028* |
| **ATQ-30, score, mean (SD)** | 57.6 (26) | 61.6 (27) | .814 | 52.1 (23.3) | .350 |
| **YFAS, score, mean (SD)** | 2.2 (1.7) | 2.5 (1.3) | .859 | 2.4 (1.5) | .819 |

**ª**Change from baseline to 8 weeks, **ªª**Change from baseline to 24 weeks; *p < .05; **p < .01; CBT = Cognitive Behavioral Therapy; BSQ-8C = Body Shape Questionnaire; K-BDI-II = Beck Depression Inventory-II in Korean; TAI = Trait-Anxiety Inventory; RSES = Rosenberg Self Esteem Scale; DEBQ = Dutch Eating Behavior Questionnaire; ATQ-30 = Automatic Thoughts Questionnaire; YFAS = Yale Food Addiction Scale.

Table MA1-8. Comparison of Calories Intake from Baseline to 8 Weeks by Intervention Condition

| **Variable** | **Control (n=25)** | **Digital CBT (n=45)** | **t** | ***p* (2-tailed)** |
| --- | --- | --- | --- | --- |
| Breakfast, kcal, mean (SD) | 162 (127.7) | 187.4 (115.3) | 0.84 | 0.403 |
| Lunch , kcal, mean (SD) | 414.4 (111.7) | 383.7 (103.9) | -1.14 | 0.258 |
| Dinner, kcal, mean (SD) | 478.8 (196.6) | 418.6 (128) | -1.53 | 0.130 |
| Snacks, kcal, mean (SD) | 208.2 (166.3) | 135.9 (86.4) | -2.38 | 0.020** |
| Total Calories, kcal, mean (SD) | 1259.3 (338.7) | 1125.8 (230.2) | -1.94 | 0.057* |

*p < .1, **p < .05; CBT = Cognitive Behavioral Therapy.

Table MA1-9. Digital CBT group had higher engagement rate compared to the control group during the intervention period.

| **Week** | **Control (n=25)** | **Digital CBT (n=45)** | ***p* (2-tailed)** |
| --- | --- | --- | --- |
| Week 1 | 96% | 100% | 0.357 |
| Week 2 | 88% | 96% | 0.341 |
| Week 3 | 84% | 89% | 0.712 |
| Week 4 | 84% | 91% | 0.443 |
| Week 5 | 76% | 93% | 0.060 |
| Week 6 | 72% | 91% | 0.046* |
| Week 7 | 64% | 89% | 0.026* |
| Week 8 | 68% | 80% | 0.383 |

*p < .05; CBT = Cognitive Behavioral Therapy.

Table MA1-10. Correlations between baseline characteristics and anthropometric measurements of 24 weeks in Digital CBT group

|  | **Body Weight Change** | | | | **BMI Change** | | | | **Body Fat Mass Change** | | | |
| --- | --- | --- | --- | --- | --- | --- | --- | --- | --- | --- | --- | --- |
|  | **8 weeks (n=38)** | | **24 weeks (n=41)** | | **8 weeks (n=38)** | | **24 weeks (n=41)** | | **8 weeks (n=38)** | | **24 weeks (n=41)** | |
| **Baseline Characteristics** | **r** | ***P*** | **r** | ***P*** | **r** | ***P*** | **r** | ***P*** | **r** | ***P*** | **r** | ***P*** |
| Weight | 0.102 | 0.541 | 0.1 | 0.535 | 0.102 | 0.543 | 0.122 | 0.449 | 0.228 | 0.170 | 0.155 | 0.332 |
| BMI | 0.128 | 0.444 | 0.003 | 0.985 | 0.137 | 0.413 | -0.011 | 0.947 | 0.203 | 0.221 | 0.049 | 0.760 |
| Fat Mass | -0.019 | 0.910 | -0.117 | 0.465 | 0.006 | 0.972 | -0.131 | 0.415 | 0.110 | 0.512 | -0.022 | 0.890 |
| Lean Body Mass | 0.093 | 0.578 | 0.189 | 0.237 | 0.075 | 0.656 | 0.226 | 0.156 | 0.246 | 0.136 | 0.313* | 0.046 |
| Motivation | -0.42** | 0.009 | -0.45** | 0.003 | -0.406* | 0.011 | -0.438** | 0.004 | -0.462** | 0.004 | -0.489** | 0.001 |
| Body Shape Satisfaction | 0.079 | 0.261 | 0.261 | 0.099 | 0.089 | 0.596 | 0.225 | 0.157 | 0.131 | 0.432 | 0.134 | 0.405 |
| Depression | 0.121 | 0.468 | 0.344* | 0.028 | 0.119 | 0.476 | 0.361* | 0.020 | 0.055 | 0.744 | 0.294 | 0.062 |
| Anxiety | 0.206 | 0.215 | 0.41** | 0.008 | 0.204 | 0.219 | 0.412** | 0.007 | 0.147 | 0.380 | 0.320* | 0.041 |
| Self-esteem | 0.165 | 0.323 | 0.468** | 0.002 | 0.174 | 0.296 | 0.455** | 0.003 | 0.008 | 0.964 | 0.323* | 0.040 |
| Restrained Eating Behavior | -0.124 | 0.459 | -0.286 | 0.07 | -0.129 | 0.441 | -0.342* | 0.028 | -0.044 | 0.794 | -0.245 | 0.123 |
| Emotional Eating Behavior | 0.051 | 0.762 | -0.049 | 0.761 | 0.047 | 0.780 | -0.102 | 0.524 | 0.096 | 0.565 | -0.088 | 0.585 |
| External Eating Behavior | -0.322* | 0.048 | -0.283 | 0.073 | -0.309 | 0.059 | -0.351* | 0.024 | -0.279 | 0.090 | -0.306 | 0.052 |
| Negative Automatic Thoughts | 0.052 | 0.758 | 0.297 | 0.060 | 0.054 | 0.749 | 0.336* | 0.032 | 0.027 | 0.872 | 0.278 | 0.078 |
| Food Addiction | 0.075 | 0.653 | 0.288 | 0.067 | 0.078 | 0.643 | 0.208 | 0.193 | 0.078 | 0.642 | 0.229 | 0.150 |
| Glucose | 0.191 | 0.250 | -0.061 | 0.703 | 0.195 | 0.242 | -0.066 | 0.684 | 0.085 | 0.613 | -0.001 | 0.997 |
| Triglyceride | -0.233 | 0.159 | -0.185 | 0.248 | -0.218 | 0.188 | -0.064 | 0.693 | -0.179 | 0.282 | -0.164 | 0.305 |
| T. Cholesterol | -0.255 | 0.122 | 0.052 | 0.748 | -0.239 | 0.149 | 0.064 | 0.692 | -0.269 | 0.102 | -0.017 | 0.916 |
| ALT | -0.123 | 0.461 | -0.258 | 0.104 | -0.121 | 0.470 | -0.223 | 0.161 | -0.035 | 0.836 | -0.259 | 0.101 |
| AST | -0.065 | 0.698 | -0.252 | 0.112 | -0.064 | 0.705 | -0.237 | 0.135 | 0.015 | 0.928 | -0.239 | 0.133 |
| GGT | -0.097 | 0.561 | -.083 | 0.605 | -0.094 | 0.575 | -0.066 | 0.681 | -0.016 | 0.926 | -0.012 | 0.939 |
| Leptin | 0.105 | 0.530 | 0.018 | 0.911 | 0.125 | 0.454 | 0.012 | 0.942 | 0.133 | 0.426 | -0.059 | 0.713 |
| Insulin | 0.058 | 0.729 | 0.085 | 0.597 | 0.060 | 0.719 | 0.124 | 0.442 | 0.185 | 0.265 | 0.114 | 0.478 |
| Insulin Resistance | 0.099 | 0.556 | 0.075 | 0.642 | 0.101 | 0.545 | 0.109 | 0.499 | 0.200 | 0.228 | 0.111 | 0.490 |

*p < .05, **p < .01; CBT = Cognitive Behavioral Therapy; BMI = Body Mass Index; Motivation, SIMS = Situational Motivation Scale; Body Shape Satisfaction, BSQ-8C = Body Shape Questionnaire; Depression, K-BDI-II = Beck Depression Inventory-II in Korean; Anxiety, TAI = Trait-Anxiety Inventory; Self-esteem, RSES = Rosenberg Self Esteem Scale; Restrained Eating Behavior, DEBQ-RE = Dutch Eating Behavior Questionnaire-Restrained; Emotional Eating Behavior, DEBQ-EM = Dutch Eating Behavior Questionnaire-Emotional; External Eating Behavior, DEBQ-EX = Dutch Eating Behavior Questionnaire-External; Negative Automatic Thoughts, ATQ-30 = Automatic Thoughts Questionnaire; Food Addiction, YFAS = Yale Food Addiction Scale; T. Cholesterol = Total Cholesterol; ALT = Alanine Aminotransferase; AST = Aspartate Aminotransferase; GGT = Gamma-Glutamyl Transpeptidase; Insulin Resistance, HOMA-IR = Homeostasis Model for Assessment of Insulin Resistance.


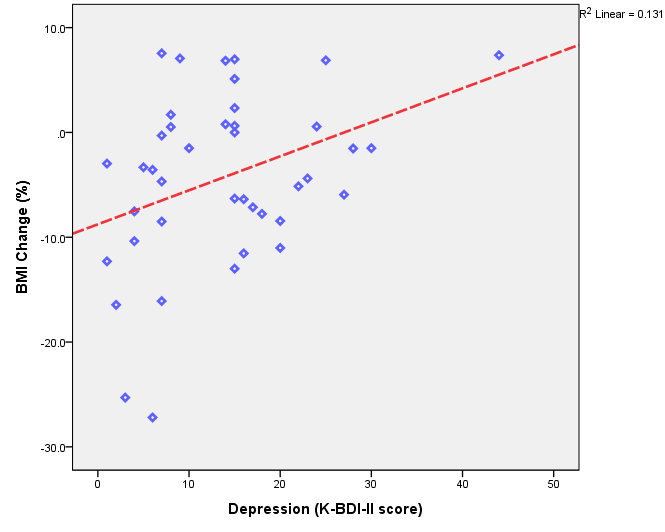

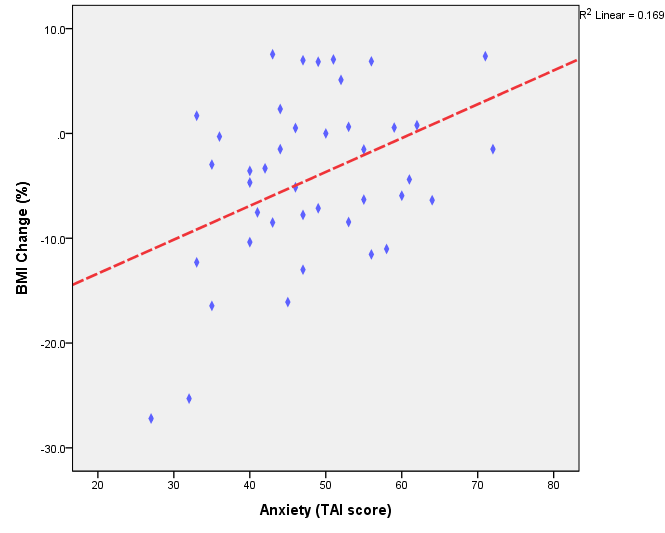

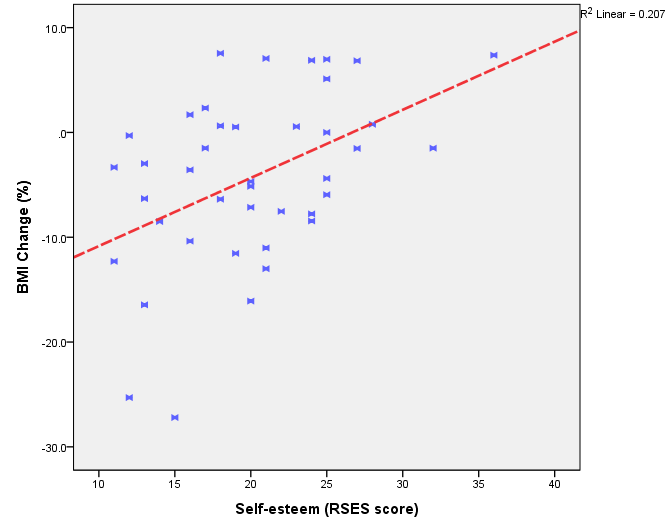

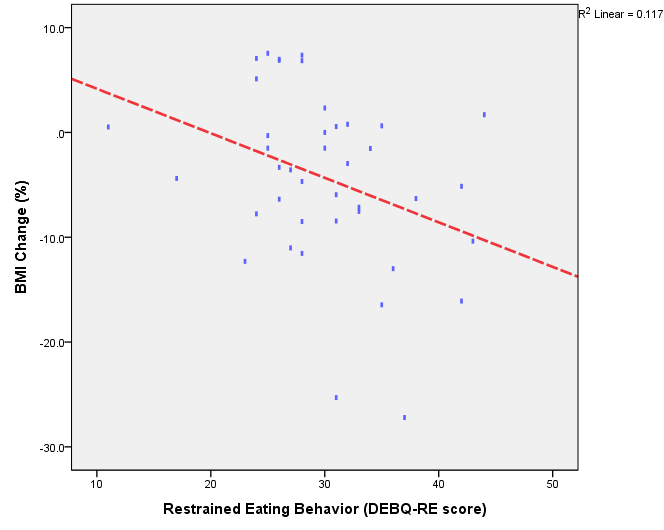

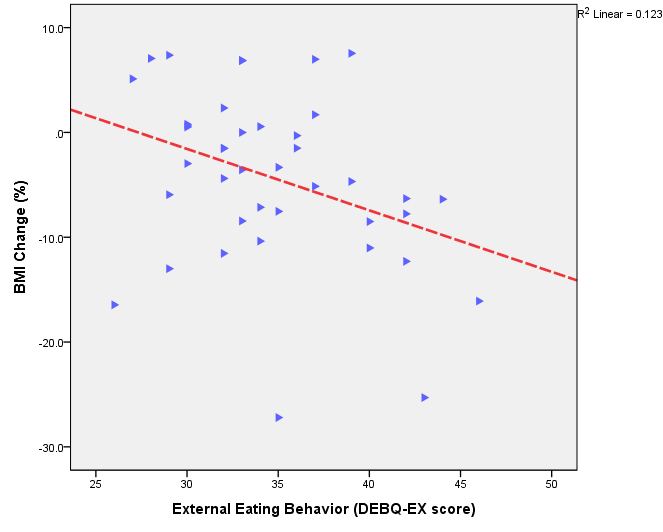

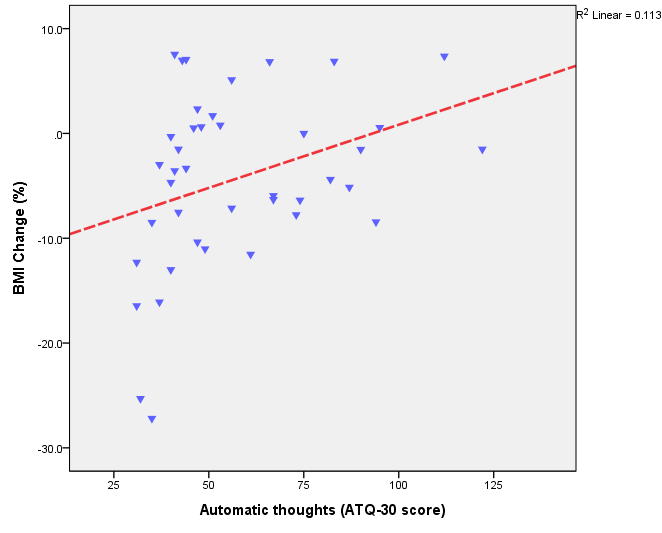


Figure MA1-1. The correlation between BMI at the long-term follow-up and the level of depression, anxiety, self-esteem, restrained eating behavior, external eating behavior, and automatic thoughts at baseline. ATQ-30: Automatic Thoughts Questionnaire; DEBQ-EX: Dutch Eating Behavior Questionnaire external eating scale; DEBQ-RE: Dutch Eating Behavior Questionnaire restrained eating scale; K-BDI-II: Korean version of the Beck Depression Inventory; RSES: Rosenberg Self-Esteem Scale; TAI: Trait Anxiety Inventory.

Table MA1-11. ROC-curve analysis for predicting efficacy of digital CBT by psychological status

|  | **ROC Analysis** | | | |
| --- | --- | --- | --- | --- |
|  | **AUC (95% CI)** | **Optimal cut off** | **Sensitivity (%)** | **Specificity (%)** |
| Weight Change (%) |  |  |  |  |
| Motivation (SIMS score) | 0.63 (0.46-0.80) | 76.5 | 59% | 74% |
| Depression (K-BDI-II score) | 0.61 (0.44-0.78) | 7.5 | 78% | 50% |
| Anxiety (TAI score) | 0.62 (0.45-0.78) | 41.5 | 87% | 41% |
| Self-esteem (RSES score) | 0.63 (0.46-0.79) | 24.5 | 35% | 91% |

ROC = Receiver Operating Characteristic; AUC = Area Under Curve; SIMS = Situational Motivation Scale; K-BDI-II = Beck Depression Inventory-II in Korean; TAI = Trait-Anxiety Inventory; RSES = Rosenberg Self Esteem Scale.
